# Skeletal muscle contraction. The thorough definition of the contractile event requires both load acceleration and load mass to be known

**DOI:** 10.1186/1742-4682-7-24

**Published:** 2010-06-18

**Authors:** Enrico Grazi, Sara Pozzati

**Affiliations:** 1 Dipartimento di Biochimica e Biologia Molecolare, Università di Ferrara, Via Borsari 46, 44100, Ferrara, Italy

## Abstract

**Background:**

The scope of this work is to show that the correct and complete definition of the system of muscle contraction requires the knowledge of both the mass and the acceleration of the load.

**Results:**

The aim is achieved by making use of a model of muscle contraction that operates into two phases. The first phase considers the effects of the power stroke in the absence of any hindrance. In the second phase viscous hindrance is introduced to match the experimental speed and yield of the contraction. It is shown that, at constant force of the load, changing load acceleration changes the time course of the pre-steady state of myofibril contraction. The decrease of the acceleration of the load from 9.8 m.s^-2 ^to 1 m.s^-2 ^increases the time length of the pre-steady state of the contraction from a few microseconds to many hundreds of microseconds and decreases the stiffness of the active fibre.

**Conclusions:**

We urge that in the study of muscle contraction both the mass and the acceleration of the load are specified.

## Background

It is a general opinion that the load (force/cross-section) determines the muscle response (power output, force and speed of contraction). Apparently it is not realized that 1. one of the components of the load, the force, is the product of the mass by the acceleration, 2. the same force is generated by an infinite number of mass and acceleration couples 3. each one of these couples displays different physical and biological effects. Let us now assume that to a muscle, of mass, m_1_, and developing the force, F_1_, is attached a load of mass, m_2_, and force, F_2_. Under these conditions the driving acceleration, a_d_, is,

Thus, at constant F_2_, changing m_2_, changes the driving acceleration, i.e. changes the time course of the pre-steady state of the contraction. This means that the characterization of muscle contraction requires the load to be defined both by its mass and by its acceleration.

We may now ask whether, in the gravitational field, it is possible to change the acceleration of the load. The answer is yes. When a muscle raises a load hanging freely, this load is associated with the acceleration of gravity. On the contrary when muscle pulls a load, rolling (in the absence of friction) on an inclined plane, the acceleration associated with the load depends on the inclination of the plane and is,

where, g, is the acceleration of gravity and, α, is the inclination of the plane. As a consequence a constant force is maintained provided that the mass is suitably changed,

A second question is whether two loads, characterized by the same force F_2_, by two different couples (m_2_' a_2_') and (m_2_" a_2_") induce the same or a different response of the muscle (power output, force and speed of contraction). In this work we try to answer the question by applying a model of muscle contraction [1,2] to the data of He et al. [3]. In short the model relates the experimental power output to the experimental speed of contraction by means of a constant, 1/k (s^-1^), that expresses the hindrance of the contractile system. As a matter of fact the actual mass and the actual acceleration of the load are unknown to He et al. [3] so we test different couples of these two parameters while keeping constant the experimental power output and the experimental speed of contraction. The result is that by decreasing the acceleration of the load the value of the constant, 1/k (s^-1^), decreases and the time length of the pre-steady state of the contraction increases. We conclude that a rigorous definition of muscle contraction requires the acceleration of the load to be known and that, at constant load force, the decrease of the acceleration of the load slows down the pre-steady state of the contraction.

## Methods

The evaluation of the effect of load acceleration on the pre-steady state of muscle fibre contraction was made on the data of He et al. [3] by making use of the model of Grazi and Di Bona [1,2].

For the convenience of the reader the model is summarized below together with an abbreviation section (Appendix 1).

The model operates into two phases. The first phase considers the effects of the power stroke in the absence of any hindrance, the result being a uniformly accelerated motion. In the second phase viscous hindrance is introduced to match the experimental speed (i.e. a uniform rate) and the experimental yield of the contraction. In short the model relates the experimental power output to the experimental speed of contraction by means of a constant 1/k (s^-1^) that expresses the hindrance of the contractile system. Data on the force - velocity curve and on the relative power outputs were taken from He et al. [3]. In the studies of He et al. [3] the isotonic contraction of rabbit skeletal muscle fibres was initiated by releasing ATP via a laser pulse from caged ATP and maintaining a constant velocity of shortening. In our model, on the contrary, muscle fibres are subjected to the load concomitantly with the activation and, after a pre-steady state, attain the isotonic contraction. Our aim is not to mimic the experiment of He et al. [3] but to feed their stationary data to our model.

We are dealing with different conditions but we see no reason why the experimental data could not fit to our model.

In the model the sarcomere is composed by n = 2000 elementary units [4], its cross-section is,(a1)

and the mobile part of the half sarcomere mass, m_1_, is,(a2)

where, ρ = 1.035 g.cm^-3^, is the density of frog sartorius muscle [5]; r = 25 nm, is the distance between the centers of two adjacent actin filaments; l_s _= 2.7 μm is the sarcomere length; 300, molecules in the tick filament; MW_M_, is the molecular mass of myosin, 520 kD [6], N, is the Avogadro's number.

### First phase

The power stroke is powered by the cleavage of ATP, 7.44 10^-8 ^pJ per molecule (E_ATP_) [7]. Power strokes occur randomly and the sequence of these events produces muscle contraction [8,9].

Energy and force delivered by the power stroke are linked by,(a3)

F_1_, is the average force over the distance *l *at the beginning of the contraction and, *l*, is the sliding of thick and thin filaments past each other, provided that E_ATP _is used up completely and that the movement occurs without hindrance.

In the presence of a load per unit area, P, the opposing force, F_2_, is.(a4)

(the factor of 2 accounts for the fact that only about half of the total cross-section of the fiber is occupied by the contractile machinery [10] and the driving acceleration is,(a5)

m_2_, is the mass of the load,(a6)

where, a_L_, is the acceleration associated with the load.

The force generated by a single power stroke is, in general, much lower than F_2 _so that it is necessary to sum up the energy delivered by the power strokes. This is possible if the frequency of the power strokes exceeds a given level, so that not all the energy provided by a power stroke is used up before the following power stroke, performed by another attached cross-bridge, occurs. This condition is fulfilled if the space, *l*_*A*_, travelled in the time between two power strokes, is lower than, *l *= E_ATP_/F_1_, so that the fraction left of the original energy, 1- |*l*_*A*_|/*l*, adds to the energy provided by the subsequent power stroke. To summarize, *l*, is constant, its value is defined by eqn. a3, where the value of F_1 _is that at the starting of the experiment. In the pre-steady state of the contraction both F_1 _and *l*_*A *_change. At the steady state both F_1 _and *l*_*A *_are constant the driving acceleration, a_d_, approaches zero. Thus bound cross-bridges make a translation of |*l*_*A*_| <*l *whose value changes with the progress of the contraction and becomes fairly constant at the steady state

The iteration procedure is as follows, The energy left after the (i-1)^th ^cycle is,(a7)

The total energy available in the i^th ^cycle is,(a8)(a9)(a10)

*l*_*A*_, the space traveled in the i^th ^cycle in the absence of hindrance, is determined by:(a11)

and the velocity, v_(i)_,(a12)

where, t_AT_, is the time between the power strokes.

Thus at any cycle the energy available, the contractile force and the driving acceleration change. In the first cycle, E_T _= E_ATP _and v = 0.

### Second phase

In the second phase the uniformly accelerated motion is converted into the uniform motion observed experimentally by introducing a viscous hindrance. To do this an hyperbolic form is assigned to the velocity, v_V_, of the masses, m_1 _and m_2_, which move under the effect of the force F_1 _[11,12],(a13)

where the reciprocal of the constant, k (s), defines the hindrance. In our system, since driving acceleration is changing at every cycle,(a14)

where, i_vv_, the increment of velocity in the time t_AT _is,(a15)

and the space, *l*_*V*_, travelled in the i_th _cycle of time length, t_AT_, is,(a16)

The total space traveled, stv, is obtained by summing up the spaces traveled in each single cycle,(a17)

Actually to avoid calculation overflow in the iterative procedure the time increment, t_I_, must be much lower than t_AT_. The value selected was t_I _= 10^-9 ^s. t_I _thus replaces t_AT _in equations (a11), (a12), (a15) and (a16) while equation (a8) is modified accordingly,(a18bis)

The hypothesis is thus made that the energy, E_ATP_, is delivered uniformly in the period t_AT_.

### Operative features

The operative features were as follows. Data on the force - velocity curve were taken from Figure six of He et al. [3], with P_0 _= 190 kN.m^-2^, a/P_0 _= 0.42, and b = 0.51 (Table 1). In the original Figure six of He et al. [3] the tension is plotted as a function of the applied shortening velocity in number of fibre length per second (ML s^-1^). We prefer, on the contrary, to plot the applied shortening velocity per half sarcomere (hsl.ML.s^-1^), where, hsl, is the half sarcomere length, as a function of the relative tension, (the actual tension, P, divided by the isometric tension, P_0_).

**Table 1:** The velocity of contraction and the ATPase rate constant as a function of the load

P/P_0_	Contraction velocity, v_v_nm.s^-1^.hsl^-1^	ATPase rate constant, s^-1^
0.00526316	1610.48	18.1578

0.0526316	1380.07	17.5316

0.105263	1172.8	16.8363

0.157895	1003.28	16.1415

0.210526	862.062	15.4473

0.263158	742.604	14.7537

0.315789	640.236	14.0606

0.368421	551.535	13.3681

0.421053	473.936	12.6761

0.473684	405.477	11.9846

0.526316	344.633	11.2937

0.578947	290.2	10.6034

0.631579	241.216	9.91354

0.684211	196.902	9.22426

0.736842	156.62	8.53553

0.789474	119.843	7.84735

0.842105	86.1343	7.1597

0.894737	55.1241	6.47259

0.947368	26.5012	5.78603

In He et al. [3] the velocity of contraction, V, number of fiber length per second (ML/s), is calculated by the equation: V = b (P_0 _- P)/(P+a), Fig. 6 of He et al. [3], where P_0 _= 190 kN.m^-2^; a/P_0 _= 0.42; b = 0.51. In this work the velocity of contraction was expressed in nm per second per half sarcomere: v_v _= hsl. ML.s^-1^, where hsl = 1350 nm. The ATPase constant was calculated from the equation, ATPase rate constant (s^-1^) = 5.1 + (18.7 × 1.94 × V)/(1+1.94 × V) where, V, is the applied shortening velocity (ML.s^-1^), 5.1 s^-1 ^is the ATPase rate constant in the isometric state, 18.7 s^-1 ^is the ATPase rate constant for shortening at infinite velocity [3].

The ATPase rate constant as a function of the applied shortening velocity were taken from Figure eight of He et al. [3] (Table 1). ATP consumption was assumed to be due only to the actin-myosin ATPase since the experiments were performed in permeabilized muscle fibers. From these data the time between the power strokes, t_AT_, was calculated as follows,(a18)

Where, n, is the number of thick filaments in the half sarcomere, 300 is the number of the myosin heads in the half sarcomere, 0.96 is the fraction of myosin heads involved in the process and, k_AT_, is the mean ATPase rate constant which changes with the load [3].

The program was operated into two steps.

a. The first step was operated in the absence of viscous hindrance and in the presence of an external load. In this step the value of the initial F_1 _was determined. Initial F_1 _is the threshold force that must be reached by the cross-bridges in order to be able to prepare the contraction. If the threshold force is not reached the system does not reach a positive driving acceleration and the contraction does not start. The right value of the initial F_1_, in a given condition, is obtained by trials, starting from a low value of F_1 _and continuing with small increments until the threshold value is reached that initiates the contraction. Although laborious the procedure is quite accurate.

b. In the second step, once the value of the initial F_1 _has been found, the value of, k (s), capable to equate the calculated velocity, v_V_, to the observed velocity, v_O_, is determined. Also in this case the search for, k (s), is made by trials. The program is stopped at v_V_/v_O _< 1.001.

## Results

In the study of muscle contraction load and muscle are usually treated as a single system. It may therefore appear a non-sense to talk about the acceleration of the load. In our model, however, load and muscle are considered as separate systems at the beginning and merge as a single system at the moment of the contraction. Under these circumstances the effects of the changes of the acceleration of the load on the contraction can be traced.

### Effect of the acceleration of the load on the initial F_1_

In this section it is shown that, at parity of the force F_2_, the decrease of the acceleration of the load (and the corresponding increase of the mass) decreases the initial F_1 _required to start the contraction.

In our previous work we considered the load as a weight (a_L _= 9.8 m.s^-2^) so, to change the load, we changed its mass [1,2]. The load, however, can be changed also by changing the acceleration while keeping constant the mass. In the following experiments the mass, m_2iso_, obtained by dividing the isometric tension per half sarcomere (2 × s_S _× P_0_) by the acceleration of gravity was used,(b1)

where, s_S_, is the sarcomere cross-section and, P_0_, is the isometric tension per unit area.

Thus the acceleration at the different loads was,(b2)

where, F_2_, is the force generated by the load.

So that, at each single load is,(b3)

where, g, is the acceleration of gravity.

It is shown that, at constant F_2_, lowering the acceleration of the load decreases the initial F_1 _to values smaller than those required when the acceleration of the load is kept at a constant, large value (9.8 m.s^-2^) and the value of F_2 _is adjusted by changing the mass. This is appreciated by comparing row by row the data of Table 2. At P/P_0 _= 0.0526, the, F_1_, required to prepare the contraction is 15.6 fold smaller when the load is defined by a large mass (1.259 10^-7 ^kg,) and a small acceleration (0.5158 m.s^-2^) than when the load is defined by a small mass (6.277 10^-9 ^kg) and a large acceleration (9.8 m.s^-2^). Thus the divergence of the two conditions increases with the decrease of the load, this is because both mass and acceleration in the two conditions are much more different at small load than at large load (Table 2).

**Table 2:** Effect of the mass and of the acceleration of the load on initial F_1_

P/P_0_	Changing mass			Changing acceleration			
	**mass Kg**	**acceleration m.s^-2^**	**Initial F_1_, pN (1)**	**mass Kg**	**acceleration m.s^-2^**	**Initial F_1_, pN (2)**	**(1)/(2)**

0.0526	6.277 10^-9^	9.8	3300	1.259 10^-7^	0.515	207	15.96

0.1579	1.988 10^-8^	9.8	31108	1.259 10^-7^	1.547	6261	4.97

0.3684	4.639 10^-8^	9.8	158060	1.259 10^-7^	3.61	85631	1.84

0.5789	7.29 10^-8^	9.8	366025	1.259 10^-7^	5.674	295915	1.24

0.7895	9.94 10^-8^	9.8	641199	1.259 10^-7^	7.7368	607261	1.055

0.9473	1.193 10^-7^	9.8	885361	1.259 10^-7^	9.284	878421	1.008

### Effect of the acceleration of the load on the pre-steady state

In this section it is shown that the decrease of the acceleration of the load (at parity of load force) delays the pre-steady state and decreases the apparent viscosity coefficient, 1/k (s^-1^).

The overall shortening of the half sarcomere under viscous regime, stv, was obtained by summing up the spaces traveled in each single cycle, stv = stv + *l*_*V*_. The information fed to the system were: P/P_0 _= 0.0526, ATPase rate = 17.5316 s^-1^, steady rate of shortening, 1380.07 nm.s^-1^.hsl^-1^, these parameters being taken from the experiment of He et al. [3]. Since the acceleration of the load in the experiment of He et al. [3] is unknown two cases were considered.

a. load acceleration, 9.8 m.s^-2^; load mass, 6.277 10^-9 ^kg. Under these conditions, to fit the P/P_0_, the ATPase rate and the steady rate of shortening of He et al. [3], F_1 _was adjusted to 3584 pN and k to 8.32 10^-5 ^s.

b. load acceleration, 0.5158 m.s^-2^; load mass, 1.259 10^-7 ^kg. Under these conditions, to fit the P/P_0_, the ATPase rate and the steady rate of shortening of He et al. [3], F_1 _was adjusted to 230 pN and k to 2.24 10^-4 ^s.

Please notice that the product of the acceleration and of the load mass is exactly the same in the two cases.

**Figure 1:**
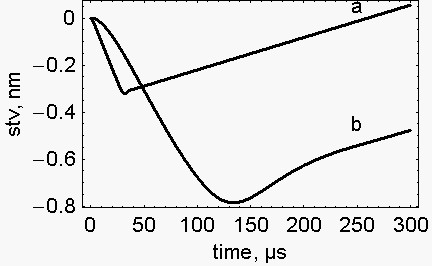
**Distance covered in the pre-steady state**. Stretching (descending limb), shortening (ascending limb). The conditions were: P/P_0_, 0.0526. Trace a. load acceleration, 9.8 m.s^-2^; load mass, 6.277 10^-9 ^kg; F_1_, 3584.474 pN, k, 8.32 10^-5 ^s. Trace b. load acceleration, 0.5158 m.s^-2^; load mass, 1.259 10^-7 ^kg; F_1_, 230 pN; k, 2.24 10^-4 ^s.

The result of the calculation is presented in Fig. 1 where the shortening of the half sarcomere under viscous regime, stv, is illustrated. The descending limb of the curves is due to the stretching by the load (negative values), the ascending limb represents the shortening due to the active reaction of the half sarcomere (negative values, shortening up to the rest length; positive values, shortening below the rest length). In curve, a, the descending limb of the curve (stretching by the load) has a length of 0.32 nm (0 - (-0.32)) and is reached in 31.7 μs. In curve, b, the descending limb of the curve has a length of 0.784 nm (0 - (-0.784) and is reached in 131 μs. (Incidentally, a similar behaviour was displayed experimentally by the tibialis anterior of the cat [4,13]). Thus, even though the load is the same, the decrease of the acceleration of the load decreases the initial F_1_, decreases the apparent viscosity coefficient, 1/k (s^-1^), delays the pre-steady state, in fact the steady rate is reached in ~30 μs in curve, a, and in ~250 μs in curve, b.

### Effects of the acceleration of the load on the initial stiffness of the active half sarcomere

In this section we show that the decrease of the acceleration of the load (at parity of load force) decreases the apparent stiffness of the half sarcomere.

An estimate of the initial stiffness of the active half sarcomere is obtained by dividing the difference between F_2 _and the initial F_1 _by stv_M_, the difference between the length of the half sarcomere at the maximum extension and the rest length.(b4)

**Figure 2:**
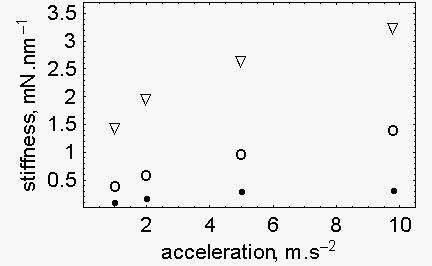
**Stiffness of the half sarcomere as a function of the acceleration of the load**. Filled circles, P/P_0 _= 0.368; open circles, P/P_0 _= 0.579; triangles, P/P_0 _= 0.789.

As it is shown in Fig. 2, the stiffness increases with the increase of P/P_0 _but it decreases with the decrease of the acceleration of the load. In fact, when the acceleration associated to the load decreases from 9.8 to 1 m.s^-2^, at P/P_0 _= 0.789 the stiffness decreases from 3.24 to 1.43 mN.nm^-1^; at P/P_0 _= 0.579 the stiffness decreases from 1.42 to 0.41 mN.nm^-1^; at P/P_0 _= 0.368 the stiffness decreases from 0.305 to 0.085 mN.nm^-1^.

## Discussion

The parameter k (s), related to the viscosity of the structure, is an important feature of the model. The viscosity originates from the interplay between water activity and the stiffness of the structure. In fact the water activity coefficient is altered by sarcomere stretching, by the cross-bridges attaching and detaching [14], by the formation of the network of filaments [15]. In turn viscosity is related to water activity since any time water activity decreases (or protein osmotic pressure increases) viscosity (and stiffness) increase. Thus muscle contraction is ruled by the interplay between water activity, viscosity and stiffness [2].

As it was shown in our previous work [1] the model also predicts a cooperative behaviour between the elementary sarcomere units. This was tested by comparing the behaviour of virtual sarcomere composed by a different number of elementary units. It was found that the value of the initial F_1 _required to initiate the contraction increased with the decrease of the number of the elementary units. This suggests that the elementary units are cooperating to overcome the load.

When presenting our model for the first time [1,2] we selected the acceleration of gravity as the load acceleration and the load was changed by changing the mass. As a result the pre-steady state of the contraction was estimated to be quite rapid, a few microseconds, thus beyond the possibility of an experimental check. We observe now that, at constant load, the time length of the pre-steady state of the contraction increases significantly with the decrease of the acceleration of the load. This time length may reach the sub-millisecond, thus allowing the prediction of our model to be tested experimentally.

He et al. [3], by measuring the steady rate of contraction and the accompanying ATPase rate, provided the values of the parameters (load, velocity of the isotonic contraction, power output) to be fed to our model. From those data and on the basis of our model we reconstructed the pre steady state of muscle contraction even though, as we stated already, our model and the muscle fibres of the experiment of He et al. [3] operate quite differently. We tacitly assumed the release of ATP from caged ATP to be immediate. The advantage of this choice is to test directly the time of reaction of the contractile apparatus, a time that appears much faster that it is usually believed. Being more realistic i.e. simulating a gradient of ATP concentration would make more complex the writing of the model but would not alter the main conclusion of this work: namely that changing the acceleration of the load changes the initial F_1 _and the time course of the pre-steady state of myofibril contraction.

At the same load, P/P_0_, 0.0526, at the same ATPase rate, 17.5316 s^-1 ^and at the same steady rate of shortening, 1380.07 nm.s^-1^.hsl^-1^, the half sarcomere is stretched differently depending on the mass and on the acceleration of the load. At the load acceleration of 9.8 m.s^-2 ^and load mass of 6.277 10^-9 ^kg the half sarcomere is stretched by 0.32 nm in 31.7 μs. At the load acceleration of 0.5158 m.s^-2 ^and load mass of 1.259 10^-7 ^kg the half sarcomere is stretched by 0.784 nm in 131 μs. Moreover, according to our model, both the initial force and the initial stiffness of the active half sarcomere decrease with the decrease of the acceleration associated to the load. These effects are the direct consequence of the equation of motion used,

where the relevance of the acceleration of the load is recognized.

**Figure 3:**
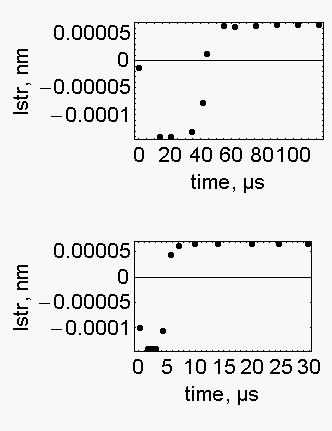
**The length of the power strokes**. The length of the power stroke was calculated by summing up the distances, *l*_*V*_, spanned in t_AT_/t_l _~ 137 consecutive cycles of the program. Upper part of the figure: P/P_0 _= 0.421; a_L _= 0.5 m.s^-2^; F_1 _= 228.43 pN; k = 2.26 10^-4 ^s. Lower part of the figure: P/P_0 _= 0.421; a_L _= 9.8 m.s^-2^; F_1 _= 2030.66 pN; k = 2.467 10^-5^s.

According to the classic view the assumed length of the working stroke is as low as 4 nm and take place in a few ms [16], in our model, on the contrary, the working stroke is much shorter and much more rapid. We define the length of the working stroke (lstr) as the distance spanned by the active sarcomere in the time between two power strokes, t_AT_. This definition may appear too wide since it includes both stretching and contraction. However there is a smooth transition between the two conditions: in the first F_1 _succumbs to F_2_, in the second F_1 _overcomes F_2_. The length of the working stroke is calculated by summing up the distances, *l*_*V*_, spanned in t_AT_/t_l _~ 137 consecutive cycles of the program. As it is shown in Fig. 3, the length of the putative working stroke first increases up to 0.00014 nm at the end of sarcomere stretching then stabilized at 0.000065 nm in the steady state. These are incredibly low figures. This is because the effect of a single working stroke is averaged between the 2000 elementary units of the sarcomere. Incidentally, the lengths of the working stroke are not influenced by the change of the acceleration of the load from 0.5 m.s^-2 ^to 9.8 m.s^-2^.

The difference between our view and the classic view is partly explained by the fact that our model tests directly the contractile apparatus while experimental tests are "contaminated" by accessory compliances that slow down the phenomenon. We report small (0.04 - 0.784 nm) and very fast (40 - 131 μs) stretching of the half sarcomere. Stretching is mostly the result of the visco-elastic distortion of the lever arm plus the compliance of the thick and thin filaments. Stretching is not *an all or none *but a relatively continuous event. So it should not be surprising that the smaller the distortion the faster the event. Our experimental settings are presently unable to record extremely short stretches. This does not means that our model is wrong.

Many reports show that the ATPase rate depends on the load [3,17-19]. No information however is available on the effect of the change of the mass-acceleration couple at constant load. Tentatively we assumed that, at constant load, muscle power output (i.e. the ATPase rate constant) and the steady rate of shortening do not change with the acceleration associated to the load. Nevertheless, owing to the very fine regulation of muscle contraction, we do not exclude that a change may occur. Perhaps the introduction of the acceleration of the load in the pre-steady could help unraveling some debated questions on muscle mechanism. It is clear however that to achieve this result the model should be confronted with experiments specifically addressed to those questions. It is sometime recognized, explicitly or implicitly, that, during acceleration, the load on the contracting muscle continuously changes because, as the mass accelerates, the force produced by the muscles changes due to force-velocity and length-tension effects. The changing muscle force in turn affects the acceleration of the load [20-22].

In general, however, in the studies on myofibril contraction very precise measurements of the force-length relationship are performed but no explicit mention of the value of the acceleration associated to the load is provided. Since the time length of the pre-steady state of the contraction is determined by the acceleration associated to the load we urge this parameter to be explicitly mentioned in the experiments.

## Conclusions

In the studies on myofibril contraction very precise measurements of the force-length relationship are performed without mentioning the value of the acceleration associated to the load.

According to our model, with the decrease of this acceleration the time length of the pre-steady state of the contraction increases up to the millisecond thus allowing our predictions to be tested experimentally.

Furthermore both the initial F_1 _(the initial force needed to start the contraction) and the initial stiffness of the active myofibril decrease with the decrease of the acceleration of the load. Thus, at constant load, the decrease of the acceleration induces the decrease of the stiffness.

All these features strongly suggest that the value of the acceleration of the load should be mentioned in all the experiments on muscle contraction.

## Competing interests

The authors declare that they have no competing interests.

## Authors' contributions

EG has made the conception and design of the manuscript; SP has contributed to the analysis and to the interpretation of the data. Both authors read and approved the final version.

## Appendix 1 - Abbreviations

a_d _driving acceleration

a_L _acceleration of the load

E_ATP _free energy of hydrolisis of one molecule of ATP in muscle

E_R _energy left after the (i-1)^th ^cycle

E_T _total energy available in the I^th ^cycle

F_1 _force of the half sarcomere

F_2 _force of the load

i number of the cycles

i_VV _increment of velocity in the time t_AT_

*l *= E_ATP_/initial F_1_

*l*_*A *_space travelled in the absence of hindrance in the time between two power strokes

l_S _sarcomere length

*l*_*V *_space travelled in the presence of hindrance in the time between two power strokes

1/k (s^-1^) hindrance of the contractile system

m_1 _mass of the mobile part of the half sarcomere

m_2 _mass of the load

n = 2000 number of the elementary units

P tension per unit area

P_0 _isometric tension per unit area

r = 25 nm distance between the centers of two adjacent actin filaments

s_S _sarcomere cross-section

stv total space travelled in the presence of hindrnce

t_AT _time between the power strokes

t_I _time length of the cycles

v velocity in the absence of hindrance

v_V _velocity in the presence of hindrance
